# LPS Counter Regulates RNA Expression of Extracellular Proteases and Their Inhibitors in Murine Macrophages

**DOI:** 10.1155/2012/157894

**Published:** 2012-03-14

**Authors:** Andreas Hald, Birgitte Rønø, Leif R. Lund, Kristoffer L. Egerod

**Affiliations:** ^1^The Finsen Laboratory, Rigshospitalet, Copenhagen Biocenter 2200 Copenhagen, Denmark; ^2^Biotech Research and Innovation Centre (BRIC), University of Copenhagen, 2200 Copenhagen, Denmark; ^3^Department of Cellular and Molecular Medicine, Faculty of Health Sciences, University of Copenhagen, 2200, Copenhagen, Denmark; ^4^Section for Metabolic Receptology and Enteroendocrinolgy, Novo Nordisk Foundation Center for Basic Metabolic Research, University of Copenhagen, 2200 Copenhagen, Denmark

## Abstract

Besides their evident importance in host defense, macrophages have been shown to play a detrimental role in different pathological conditions, including chronic inflammation, atherosclerosis, and cancer. Regardless of the exact situation, macrophage activation and migration are intimately connected to extracellular matrix degradation. This process is accomplished by multiple proteolytic enzymes, including serine proteases and members of the matrix metalloproteinase family. In this study, we have utilized qPCR arrays to simultaneously analyze the temporal expression pattern of a range of genes involved in extracellular matrix metabolism in the mouse derived-macrophage cell line RAW 264.7 following stimulation with LPS. Our results revealed that LPS induces the expression of matrix metalloproteinases while at the same time decreased the expression of matrix metalloproteinase inhibitors. The opposite scenario was found for the genes encoding serine proteases, which were downregulated while their inhibitors were upregulated. In addition, intergenic comparison of the expression levels of related proteases revealed large differences in their basal expression level. These data highlight the complexity of the gene expression regulation implicated in macrophage-dependent matrix degradation and furthermore emphasize the value of qPCR array techniques for the investigation of the complex regulation of the matrix degradome.

## 1. Introduction

Macrophages are involved in many aspects of immunity. In addition to clearing apoptotic cells and cellular debris following infection and tissue damage, they also play an important regulatory role by modulating immune responses through the secretion of pro- or anti-inflammatory cytokines [1]. Though macrophages clearly are important for a functional immune system, they have also been shown to exacerbate pathological conditions involving chronic inflammatory reactions, including atherosclerosis and cancer [2–4].

The participation of monocytes/macrophages in an immune response depends on their ability to migrate through the tissue to the inflammatory site, and it has been thoroughly documented that this process involves degradation of the extracellular matrix (ECM) [5–7]. For this purpose, macrophages may orchestrate the recruitment/activation of cells capable of secreting the necessitated proteases or they may produce the proteases and protease-activators themselves [8].

Seemingly, the serine protease, plasmin, and members of the plasminogen- (Plg) activation system (PA-system) are of particular importance for monocyte/macrophage migration, as mice deficient in genes encoding either Plg- or a PA-system component display a severe impediment in macrophage recruitment [5, 9]. Though macrophages do not produce Plg themselves, they express urokinase-Plg activator (uPA) and its cell surface receptor, uPA-receptor (uPAR), thus facilitating a localized conversion of Plg to active plasmin [5]. Besides the PA-system, macrophage migration has also been shown to depend on individual matrix metalloproteinases (MMPs), such as MMP9 and MMP12 [9, 10]. The protease substrates, which need to be degraded prior to an adequate recruitment of macrophages, have yet to be identified. This task is not only hampered by the complexity of the ECM, but also by the fact that global datasets describing the temporal expression of the genes involved in ECM metabolism are lacking.

In the presented study, we have determined the temporal changes in the expression of genes encoding key proteases and ECM components in macrophages following activation by LPS using the StellARray qPCR array system, which allows for the simultaneous quantification of up to 95 gene transcripts. The qPCR data were analyzed using both a recently published Global Pattern Recognition (GPR) algorithm [11] and a standard fold-change analysis. In addition, the mRNA level of all analyzed genes was directly compared using a novel method in which DNA was used as a global calibrator for all genes. Utilizing these methods, we were able to identify a pronounced difference in the basal RNA expression level of related proteases (e.g., a 16-fold higher basal expression level was observed for MMP13 versus MMP8 and for MMP9 versus MMP2). Furthermore, the Plg activators and their inhibitors were shown to be inversely regulated by LPS stimulation, which decreased the RNA expression of the activators while the RNA expression of the inhibitors was increased. A similar but opposite pattern was found for MMPs where RNA expression was increased for MMPs, whereas it was decreased for the MMP inhibitor Tissue Inhibitor of MMPs- (Timp-) 2. Thus, stimulation with a single compound induces inverse regulation of genes with opposing functions.

## 2. Materials and Methods

### 2.1. RAW 264.7 Cells

The mouse-derived macrophage cell line RAW 264.7 was propagated in culture medium defined as DMEM, containing 10% FCS and 1% Penicillin-Streptomycin (Sigma-Aldrich, Brøndby, Denmark). Experimental cell cultures were set up in 6-well culture plates by seeding 5 × 10^5^ cells in 4 mL culture medium in each well. The following day, the culture medium was exchanged with serum-free DMEM. After 4 hours, vehicle (PBS) or LPS (Sigma-Aldrich, Brøndby, Denmark) was added to appropriate cultures to a final concentration of 100 ng/mL. After 0, 2, 6, and 18 hours of vehicle or LPS stimulation, the RAW 264.7 cultures were harvested by removing the supernatant and lysing the cells using the NucleoSpin RNAII kit for RNA purification (Macherey-Nagel, Germany). The lysates were stored at −80°C.

### 2.2. RNA Purification and Reverse Transcription

RNA from RAW 264.7 cell cultures was purified using the NucleoSpin RNAII kit (Macherey-Nagel, Düren, Germany). During the purification procedure, samples were treated with the DNase included in the kit according to manufactures instruction. The RNA concentration of the final eluate was determined using a NanoDrop1000 (Thermo Scientific, Copenhagen, Denmark).

Reverse transcription was performed using the SuperScript II RT-PCR system (Invitrogen, Nærum, Denmark) with random primers (Roche Applied Science, Hvidovre, Denmark) and dNTPs (Invitrogen, Nærum, Denmark). cDNA was transcribed using 1 *μ*g RNA in a total reaction volume of 20 *μ*L.

### 2.3. qPCR Array Analysis

The custom designed StellARray qPCR arrays (Lonza, Basel, Switzerland) were utilized according to the manufactures instruction. Briefly, 2.1 mL qPCR master mix was prepared using 1050 *μ*L 2X SYBR Premix Ex Taq (TaKaRa, Shiga, Japan), 1046 *μ*L water and 4 *μ*L of the above-described cDNA solution. 20 *μ*L was added to each well of the StellARray 96 well plate (one plate for each sample). In addition, three samples of mouse genomic DNA were analyzed on similar plates using a final concentration of 0.2 ng/*μ*L DNA (Zyagen, California, USA). A LightCycler480 (Roche Applied Science, Hvidovre, Denmark) was used with the following thermal profile: 5 min at 50°C, 30 sec at 95°C, 40X (30 sec at 95°C, 1 min at 60°C). Melting curve analysis was performed to test the specificity of the primer pairs. The Cp's were obtained using the Second Derivative Maximum Method using the LightCycler480 software. The plate setup, listing the analyzed genes, is presented in supplementary material Table S1 available online at doi: 10.1155/2012/157894. 

To enable direct comparison of RNA levels from different genes, a genomic DNA sample was used as calibrator and the data was further normalized using a GeNorm-derived normalization factor based on Ywhaz, Gapdh, and Tbp [12]. Importantly, when calculating fold changes of individual genes using these relative RNA values, the results are equal to a normal delta delta Cp calculation since the DNA in this case only functions as a calibrator sample and consequently does not influence the fold changes observed between two samples [13]. Furthermore, the use of DNA as an intergenic calibrator requires that the compared genes are equally represented in the genome. In the current set of investigated genes a BLAST search of the amplified regions have shown that only the reference genes RN18s, Gapdh, and Ywhaz were duplicated thus disqualifying these for normalization to DNA. Finally, the use of DNA as a calibrator is evidently only possible when the primer pairs are not intron spanning:

(1) $$\text{Relative}\;\text{Copy}\;\text{Number}\frac{2^{-(\text{Cptarget}-\text{CpDNA})}}{\text{NF}}\times C,$$

where NF designates a GeNorm derived normalization factor based on Ywhaz, Gapdh and Tbp, and where Cp designates a threshold values derived using the Second Derivative Method, and *C*  is an arbitrary constant dependent on the DNA concentration. In this study  *C* = 1000.

Besides a standard delta delta Cp-based fold change analysis, the data were analyzed using the GPR algorithm software supplied by the distributor of the StellARray kits (Lonza, Basel, Switzerland). Shortly, the GPR algorithm first calculates delta Cp values for each gene of interest in comparison with all other analyzed genes (normalizers). For each gene-normalizer combination, the delta Cp values for the experimental and control group are compared using a standard  *t*-test. For every gene, a “hit” is recorded for each obtained *P*-value less than 0.05. Next, all genes are given a score based on the number of hits and ranked accordingly. Finally, a bootstrap analysis is used to convert the individual scores to *P*-values [11].

In addition to the GPR *P*-values, the software also calculates fold changes in gene expression based on normalization to either 18sRNA (RN18s fold change value) or by normalization to ten genes selected by the software using an unpublished algorithm (GPR fold change value).

## 3. Results

### 3.1. The Effect of LPS on Gene Expression in Macrophages Is Reliably Recapitulated by Array Analysis

Cultures of RAW 264.7 cells were treated with 100 ng/mL LPS or vehicle and harvested after 2, 6, or 18 hours. The mRNA expression levels of 95 genes were analyzed by StellARray qPCR array. A sample of genomic DNA containing all of the genes encoding the assayed transcripts was used as a calibrator and Tbp, Ywhaz, and Gapdh transcripts were further used to generate a GeNorm-based normalization factor to correct for inter-assay variations. The *M* values for Tbp, Ywhaz, and Gapdh were between 0.184 and 0.265 indicating that the three genes were suitable as reference genes [12]. The relative expression levels for all analyzed genes are presented in Table S2.

The ability of the precoated qPCR arrays to generate trustworthy Cp values was assessed by comparing expression changes for a number of genes (Mmp9, Mmp12, Mmp13, Gapdh, Tbp) using the same cDNA for both the arrays and for conventional single gene expression analysis using SYBR green-based qPCR. No differences were found in the data derived from the two different methods, thus showing that the arrays delivered trustworthy results of a high standard (data not shown).

**Figure 1:**
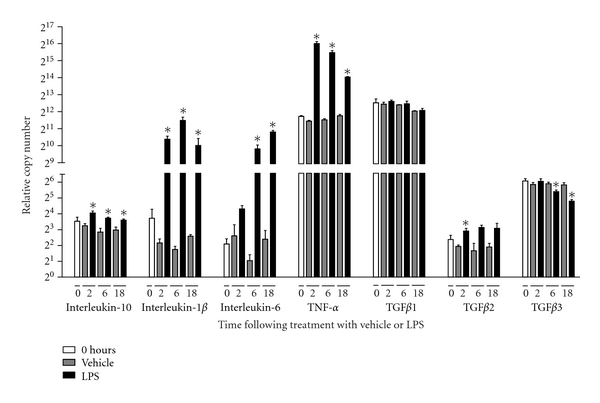
LPS induced RNA expression changes can be determined by StellARray qPCR arrays. RAW 264.7 cell cultures were stimulated with 100 ng/mL LPS or vehicle for 0, 2, 6, and 18 hours, at which time point RNA was harvested for sequential gene expression analysis. LPS stimulation led to a change in gene expression levels up to 512 fold (Il-6). This effect was already evident after two hours and was sustained for at least 18 hours. Pronounced differences in the basal level of expression (0 hours) were observed between genes (e.g., TGF*β*1 and TGF*β*2). Data were analyzed by a two-tailed Mann-Whitney test. *P < 0.05 between LPS and vehicle-stimulated cultures. *n* = 4 for all groups.

To confirm that the LPS stimulation of RAW 264.7 cells had led to the expected inflammatory response, we scrutinized the mRNA expression of classical inflammatory cytokines. The data showed that upon LPS stimulation, RAW 264.7 cells responded by increasing the expression of interleukin (Il)-1*β*, Il-6, Il-10, TNF-*α*, and TGF*β*2, while TGF*β*1 expression was unaffected and TGF*β*3 expression decreased over time. It was noted that the basal expression level of the cytokines varied considerably at 0 hours, at which point the expression of TNF-*α* and TGF*β*1 was some 256-fold higher than that of Il-1*β*, Il-6, and Il-10. In comparison with LPS treatment, the mRNA expression level of the cytokines varied only to a minor degree over time following vehicle treatment (Figure 1).

### 3.2. LPS Increases the RNA Expression of MMPs and Decreases the Expression of MMP Inhibitors

**Figure 2:**
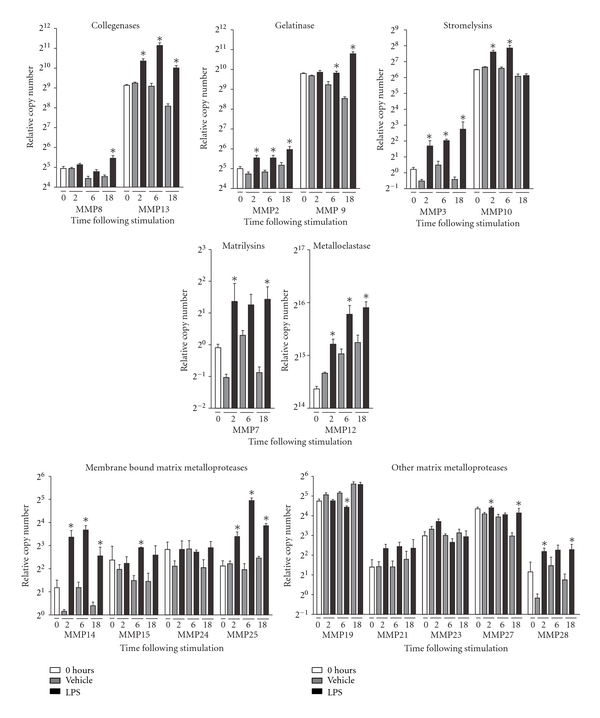
Widespread increase in the MMP gene expression level following LPS treatment. The RNA expression of MMPs following LPS treatment of macrophage cultures was generally increased compared to vehicle stimulated cultures. The basal expression level of MMPs (0 hours) varied considerably and the most abundantly expressed MMP (MMP12) had a 20,000-fold higher expression level than the lowest detectable MMP (MMP3). Data were analyzed by a two-tailed Mann-Whitney test. *P < 0.05 between LPS and vehicle stimulated cultures. *n* = 4 for all groups.

A comparison of the relative basal RNA expression levels of MMPs in untreated RAW 264.7 cultures revealed that the expression levels of MMP genes varied considerably even within the same classes of MMPs. For instance, the collagenases, MMP8 and MMP13, showed a 16 fold difference in expression levels, and the gelatinases, MMP2 and MMP9, showed a 32-fold difference in expression levels (Figure 2). A comparison of LPS and vehicle-stimulated RAW 264.7 cultures showed that LPS treatment led to a general increase in MMP RNA expression with few exceptions (Figure 2). When comparing vehicle-treated cultures with cultures harvested at 0 hours, it was clear that either serum deprivation or the time span lasting up to 18 hours had an effect on MMP RNA expression levels, which in most cases were decreased. However, this fact had no evident effect on the ability of LPS to increase the MMP RNA level.

**Figure 3:**
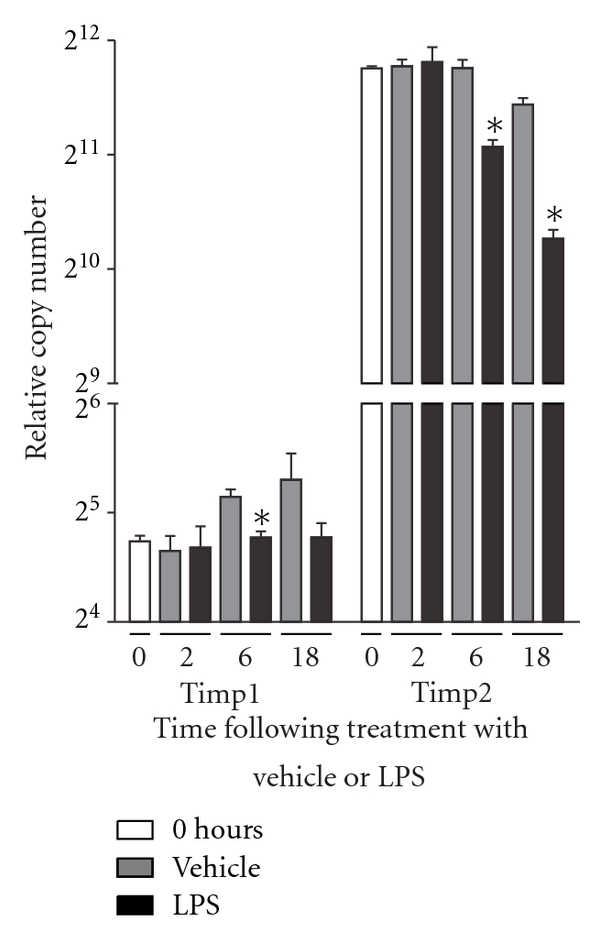
The RNA expression of genes encoding MMP inhibitors are downregulated in activated macrophages. Whereas LPS led to a general increase in MMP gene expression, the genes encoding the MMP inhibitors TIMP1 and TIMP2 were downregulated in LPS-stimulated cultures compared to vehicle-treated control cultures. The difference in the basal RNA expression levels (0 hours) between Timp1 and Timp2 differed by 128 folds. Data were analyzed by a two-tailed Mann-Whitney test. *P < 0.05 between LPS and vehicle-stimulated cultures. *n* = 4 for all groups.

As actual MMP activity is strictly regulated by TIMPs, we chose also to investigate the effect of LPS stimulation on TIMP RNA expression using the StellARray qPCR array system. We found that the general increase in MMP RNA expression following LPS treatment was accompanied by a decrease in the RNA expression of the MMP inhibitor TIMP2. TIMP1 RNA expression, however, remained fairly constant over time, though a small decrease following LPS treatment was observed at 6 hours post-treatment compared to vehicle-stimulated controls. Furthermore, the Timp1 gene was expressed at levels some 128-fold lower than Timp2 (Figure 3). Timp3 was not found to be expressed in neither LPS nor vehicle-stimulated cultures. In contrast, Timp4 was expressed at low levels but not regulated by LPS treatment (data not shown).

### 3.3. Decreased RNA Expression of Plg-Activators Is Accompanied by an Increased Expression of Plg-Activator Inhibitors

**Figure 4:**
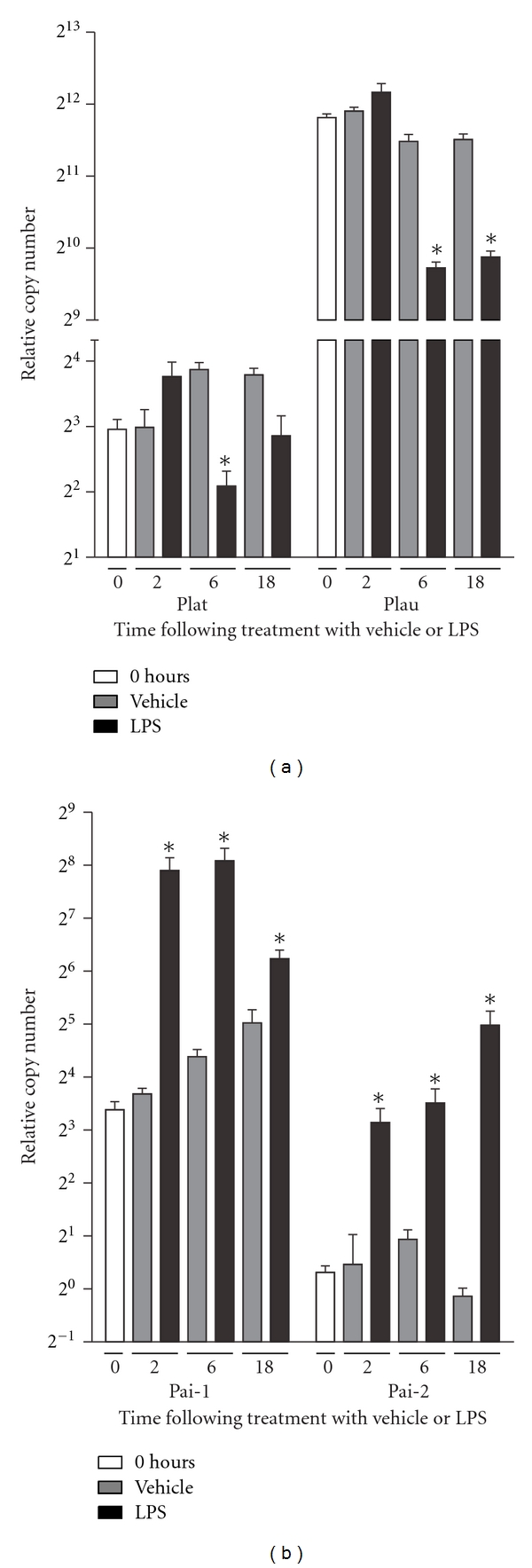
The RNA encoding plasminogen activators are downregulated while their inhibitors are upregulated. (a) Six hours following LPS stimulation, RAW 264.7 cells downregulated the expression of genes encoding the two main Plg activators, tPA and uPA. (b) In contrast to the decreased expression of Plat and Plau, the expression of the two tPA and uPA inhibitors, Pai-1 and Pai-2, was increased by up to 16 folds. Data were analyzed by a two-tailed Mann-Whitney test. *P < 0.05 between LPS and vehicle-stimulated cultures. *n* = 4 for all groups.

Following LPS treatment, RAW 264.7 cells downregulated their expression of the two Plg-activators Plat and Plau (tPA and uPA) by two and four folds, respectively. However, this effect was first detectable at 6 hours following treatment. Further analyses of the relative RNA expression levels between Plat and Plau, revealed that Plau was expressed at levels that were approximately 512 fold higher than Plat (Figure 4(a)).

While the RNA expression of the Plg-activators was decreased by LPS treatment, the opposite was true for the inhibitors of tPA and uPA, PAI-1, and PAI-2 (Serpine1 and Serpinb2). Their expression was increased by up to 32 folds. This was evident already at two hours following treatment. In addition, the direct comparison of Serpine1 and Serpinb2 showed that the relative expression level of Serpine1 was 8 fold higher than that of Serpinb2 (Figure 4(b)).

### 3.4. Genes Encoding Extracellular Matrix Proteins Are Not Widely Expressed by RAW 264.7 Cells

Extensive analysis of the expression of genes encoding ECM components revealed that this group of genes was expressed at a very low level with few exceptions, such as Fn1, Lama5, and especially Lamc1, which had a basal expression level that were approximately 100 fold higher than that of the other detectable gene transcripts. Among the gene transcripts that could be detected, only Vtn, Lama2, and Lamb3 seemed to be affected by LPS treatment, in that they were increased by two to 16 fold (Figure S1).

### 3.5. Other Protease Systems Expressed by RAW 264.7 Cells

In addition to the MMPs and the PA-system, the RNA expression of a number of proteases not belonging to these groups were investigated. We found that the Tmprss6, Prss8, and St14 genes, encoding transmembrane protease serine 6, Prostasin, and Matriptase, respectively, were expressed by RAW 264.7 cells. In addition, St14 RNA expression was found to be downregulated two fold by LPS treatment (Table S2).

### 3.6. Global Pattern Recognition Analysis

The GPR algorithm published by Akilesh et al. (2003) is made available by Lonza (Lonza, Switzerland) to facilitate the analysis of data derived from their qPCR array system, StellARray [11]. Without the use of prior-defined reference genes, the GPR algorithm analyzes for differential gene expression patterns between two groups, generating a *P*-value based on the consistency of the biological replicates. We analyzed the RAW 264.7 expression data using the GPR algorithm on the following datasets: LPS versus vehicle after 2, 6, and 18 hours. The GPR algorithm identified significant changes in the expression pattern of many of the genes that were also found to be up- or downregulated as determined by classical fold change analysis. However, a number of genes with a seemingly constant expression level, according to both GPR fold change analysis and classical fold change analysis, also received low GPR *P*-values (e.g., TGF*β*1) (Table S3). These data illustrate the different meanings of GPR derived *P*-values and fold-change derived *P*-values and emphasize that there is no direct link between changes in gene expression levels and GPR *P*-values. Thus, GPR *P*-values cannot be applied to fold change data for the individual gene transcripts.

## 4. Discussion

In the present study, we successfully implemented a novel method for intergenic comparison of RNA transcript levels using genomic DNA encoding all assayed transcripts as a calibrator. Using this method, we revealed considerable differences in the relative levels of gene transcripts between various protease systems and ECM components. Furthermore, we found that a group of genes involved in ECM metabolism was inversely regulated in macrophages following stimulation with a single compound, LPS. Interestingly, this inverse regulation could function to induce a synergistic effect on ECM turnover or simply reflect the complex and tight regulation of extracellular proteolysis, as it was mainly genes encoding proteases or protease activators and their inhibitors that were counterregulated. The observed alterations in gene expression levels over time in vehicle-stimulated cultures likely reflect serum deprivation or a time dependent change in the expression pattern. In any case, it did not block the proinflammatory effects of LPS treatment. Finally, we evaluated and compared a novel GPR analysis tool for assessing transcriptional changes in array data with a classical delta delta Cp-based fold change analysis. 

To gain an in depth knowledge of protease gene transcription in RAW 264.7 cells before and after LPS stimulation, we determined the relative expression levels using a DNA calibrator. Alternatively, absolute qPCR could have been applied for the direct comparison of transcript numbers of individual genes. However, this method relies on standard curves with known copy numbers generated using *in vitro* transcribed sense RNA, plasmids or oligonuclotides. This input material needs prior normalization to weight, total RNA, size or cell number, which may vary independently of target gene regulation and thereby increase the risk of skewing data [14].

The use of genomic DNA as a global calibrator for all genes requires that all genes are equally represented in the genome, and if not (such as genes located on the sex chromosomes or duplicated genes), a correction for the copy number must be performed. In the current study, only Timp1 was found to be located on a sex chromosome and consequently one cycle was subtracted from the DNA calibrator Cp value for this gene. Furthermore, only the reference genes included (Ywhaz, Gapdh, Rn18s) had verified gene duplications, whereas none of the target sequences showed any potential of being represented more than once in the genome. In addition, melting curve analysis showed that the used primers for each gene only gave rise to a single product. Thus, using a DNA sample as a calibrator we obtained an equal arbitrary set point for RNA transcript levels. This method provides a simple way for intergenic RNA transcript comparisons, though extra caution for DNA contamination should be taken. Despite the potential pitfalls, the intergenic differences we found using this method are corroborated by other studies, which have performed absolute RNA quantification analysis of MMP related genes in unstimulated human monocytes [15]. In this context, we found MMP9, MMP10, MMP19, and TIMP2 to be highly expressed in comparison to MMP2, MMP3, MMP21, and TIMP3, which mimics the data presented by Reel et al., 2011 [15]. However, it should be noted that differences were also observed which likely could be addressed to differences in the model systems.

It is well established that macrophages, upon activation, change the gene expression levels of many of the components involved in ECM degradation [16]. The effect of LPS on the RAW 264.7 macrophage transcriptome has previously been investigated using DNA chip technology [17]. In comparison to this whole genome chip analysis, we identified an additional 20 protease/ECM related genes with an altered expression pattern [17]. This possibly reflects the high sensitivity of qPCR arrays or varying levels of macrophage activation between the two studies. Both studies identified PAI-1 and PAI-2 as being highly upregulated on the RNA level, however, Gao et al. found TIMP-1 to be upregulated whereas the current analysis only showed a minor effect of LPS treatment on TIMP-1 RNA expression [17]. In addition, our expression data is corroborated by studies of LPS-treated human-derived monocytes, in which increased levels of MMP9, MMP10, MMP14, and decreased levels of TIMP-2 were reported [15]. LPS treatment of isolated tissues, containing a mixed cell population including macrophages, also leads to increased MMP and decreased TIMP2 RNA expression levels [18]. Interestingly, the latter study also reported altered expression of genes that were not affected by LPS treatment of RAW 264.7 cells in the current study. This may be due to direct or indirect induction of gene transcription in other cell types (e.g., fibroblasts) or reflect the importance of the microenvironment in terms of regulating gene expression in macrophages.

The protein expression of extracellular proteases and their inhibitors is regulated at several levels, including the induction of transcriptional activity and by the modulation of mRNA turnover [19]. However, changes in the mRNA levels of extracellular proteases does not directly reflect changes in the extracellular proteolytical environment as many extracellular proteases and their activators are secreted as inactive proenzymes, which themselves need to be proteolytically activated. In addition, in an *in vitro* system such as the one used in the current report, the protease activators needed to activate the proteases produced by LPS-stimulated RAW 264.7 cells may be entirely absent. Despite these facts, *in vivo* studies have shown that for some proteases, in defined tissues, mRNA and protein expression levels and even proteolytical activity correlates [20, 21]. However, in other situations, such as the secretion of preformed MMP9 by neutrophils following a proinflammatory stimulus, there is no direct link between protein synthesis and secretion [22, 23]. These facts emphasize that expression arrays of ECM remodulation genes do not in all cases reflect the actual proteolytical environment. However, highly sensitive qPCR arrays limited to proteases and ECM components, as the one presented in the current report, are a valuable tool for investigating the signaling pathways leading from extracellular stimuli to gene expression on the RNA level. The signaling pathway from LPS stimulation of macrophages/monocytes to MMP gene transcription is highly complex but has been shown to depend on MAP kinases *in vitro*, while *in vivo*, a pathway involving cyclooxygenase activation and prostaglandin synthesis followed by cAMP production also is involved [15, 24]. In the current study, we found that macrophage activation by LPS led to a general increase in MMP RNA expression levels though to a varying extent. This likely reflects differences in the promoter sequences of MMP genes [25] or in the pace at which the individual mRNAs are degraded. The surprising observation that LPS had opposite effects on RNA expression levels of genes encoding proteases and inhibitors belonging to the same protease system (e.g., in the PA-system, uPA was downregulated and PAI-1 and PAI-2 were upregulated) could also reflect variations in the promoter sequences or in mRNA degradation, which would serve to enhance the tight regulation of Plg activation.

The fact that extracellular proteases have overlapping functions emphasizes the value of the simultaneous investigation of multiple proteases as performed in the present study. This may be exemplified by the current array data showing that macrophages express both of the two Plg activators tPA and uPA, which is corroborated by previous reports [26, 27]. In addition, our array data reveal that macrophages express tPA at a 4000 fold lower level than that of uPA on the RNA level. High-resolution arrays as the one presented may also prove valuable in complex disease models where an interaction or a direct functional overlap between different proteases is presumed to play a role [28, 29].

**Figure 5:**
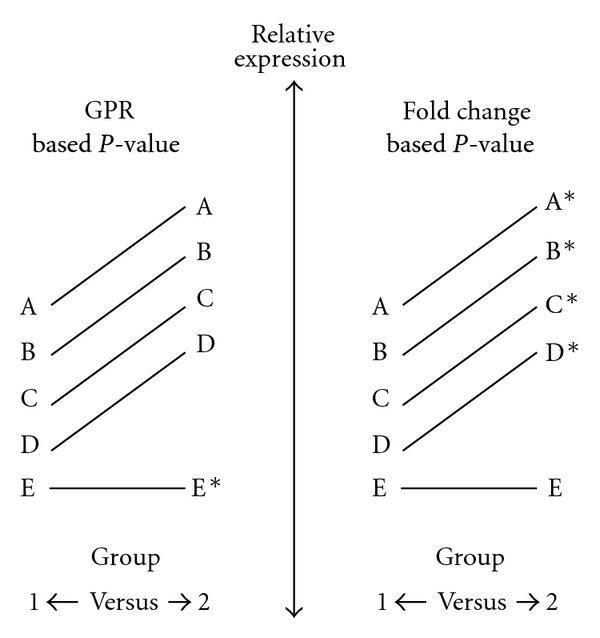
Schematic diagram of expected  *P*-values based on fold change and GPR analyses. The left side of the diagram shows a hypothetic GPR analysis of two groups (1 & 2) with a general and equal increase in gene expression levels in group 2 compared to group 1. In this case it is the single gene “E” that does not change its expression level that receives the lowest  *P*-values. This result is based on the GPR algorithm where all genes are normalized to each other. The right side of the diagram shows the same scenario but analyzed using a classical fold change statistical analysis (e.g., Mann-Whitney test). As depicted, these two methods, although both mathematically consistent, give rise to different results. * indicates a statistical significant change.

The StellARray qPCR platform proved to be an efficient method for obtaining expression data and the GPR *P*-value, based on the GPR algorithm, is an interesting approach for qPCR array analysis as there is no need for predefined reference genes [11]. However, as the GPR algorithm is a pattern-based analysis software, which is generally visualized by comparing the relative “position” of each gene in one group to its “position” in the other group (Figure 5), it should be kept in mind that the GPR *P*-value does not provide any information regarding whether the expression level of the individual gene has been altered. The presented data corroborate this notion as GPR *P*-values in some cases were below 0.05 for genes with minute and insignificant changes in expression levels as determined by a classical delta delta Cp analysis. As the GPR algorithm does not provide information regarding the significance of up-downregulation, the biological interpretation of GPR *P*-values is difficult and care should be taken not to confuse GPR *P*-values for *P*-values based on actual fold change analyses. Thus, it should be stressed that the presented GPR fold change values and the RN18s-based fold change values determined by the GPR software cannot be combined with the GPR *P*-values. In relation to the GPR fold change values, we found that they were highly similar to the values obtained using a GeNorm-derived normalization factor based on Ywhaz, Gapdh, and Tbp. In comparison, the Rn18s-based fold change values were a bit skewed compared to both of these values emphasizing that single reference gene normalization is jeopardized by potential regulation of the reference gene [30].

In conclusion, the presented data and analyses show how activated macrophages respond to LPS by altering the balance of gene expression levels between extracellular proteases and their inhibitors. Furthermore, by taking advantage of a genomic DNA calibrator, we were able to show that the relative gene expression levels between related genes differ with up to 500 folds. The presented approach may not only serve as a highly sensitive expression screening but also be used to directly investigate signaling pathways and gene expression regulation on a higher level.

## Supplementary Material

Table S1: To analyze the effect of LPS on gene expression in RAW 264.7 cells we customized a qPCR StellARray plate from Lonza, Switzerland. The primer pairs included on the plate were specific for the listed genes, which include inflammatory mediators, reference genes, and genes involved in the regulation of extracellular proteolysis.
Table S2: The expression of selected genes in RAW 264.7 cells before and after (2-18 hours) stimulation with LPS. The values present the relative expression following normalization to DNA as described in the materials and methods section.
Table S3: Gene expression in LPS and vehicle stimulated RAW 264.7 cultures was analyzed after 2, 6, and 18 hours. The GPR fold change (Fold) is presented and GPR p-values (p-value) are highlighted in red when below 0.05. The GPR fold change values are provided in this table to indicate how the gene is regulated, but it should be stressed that the GPR p-values are not directly linked to the fold change values.
Figure S1: The graph depicts the expressional changes of genes encoding extracellular matrix components. Gene expression levels are normalized to DNA as previously described. Data were analyzed by a two-tailed Mann-Whitney test. ∗p<0.05 between LPS and vehicle-stimulated cultures. n= 4 for all groups.

## Abbreviations

ECM: Extracellular matrix
MMP: Matrix metalloproteinase
GPR: Global Pattern Recognition
(PA)-system: plasminogen activation system.
