# Supplementary material for: LPS Counter Regulates RNA Expression of Extracellular Proteases and Their Inhibitors in Murine Macrophages
Source: Mediators Inflamm. 2012 Mar 14;2012:157894. doi: 10.1155/2012/157894 (PMC3317238; doi:10.1155/2012/157894)
Supplement: Supplementary file 1 — Table S1: To analyze the effect of LPS on gene expression in RAW 264.7 cells we customized a qPCR StellARray plate from Lonza, Switzerland. The primer pairs included on the plate were specific for the listed genes, which include inflammatory mediators, reference genes, and genes involved in the regulation of extracellular proteolysis. Table S2: The expression of selected genes in RAW 264.7 cells before and after (2-18 hours) stimulation with LPS. The values present the relative expression following normalization to DNA as described in the materials and methods section. Table S3: Gene expression in LPS and vehicle stimulated RAW 264.7 cultures was analyzed after 2, 6, and 18 hours. The GPR fold change (Fold) is presented and GPR p-values (p-value) are highlighted in red when below 0.05. The GPR fold change values are provided in this table to indicate how the gene is regulated, but it should be stressed that the GPR p-values are not directly linked to the fold change values. Figure S1: The graph depicts the expressional changes of genes encoding extracellular matrix components. Gene expression levels are normalized to DNA as previously described. Data were analyzed by a two-tailed Mann-Whitney test. ∗p<0.05 between LPS and vehicle-stimulated cultures. n= 4 for all groups. [file 157894.f1.pdf]

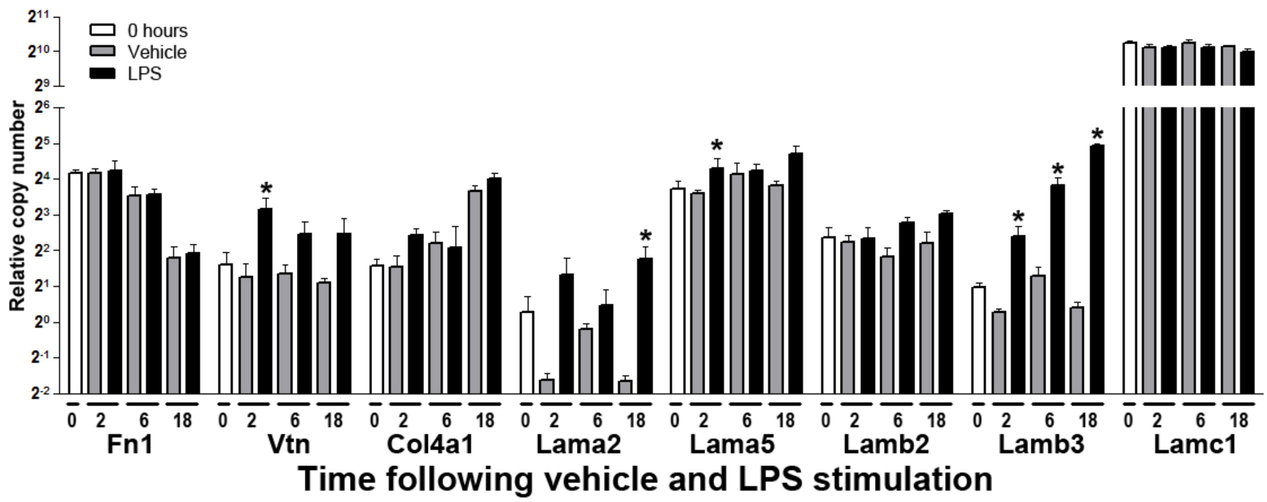

Figure S1. Relative expression levels of genes encoding ECM components

Most of the investigated genes related to ECM production were found not to be expressed by macrophages. However, of those that were, a few were also upregulated following LPS stimulation.
