# Supplementary material for: LPS Counter Regulates RNA Expression of Extracellular Proteases and Their Inhibitors in Murine Macrophages
Source: Mediators Inflamm. 2012 Mar 14;2012:157894. doi: 10.1155/2012/157894 (PMC3317238; doi:10.1155/2012/157894)
Supplement: Supplementary file 2 [file 157894.f2.pdf]

Cytokines	MMPs	MMP inhibitors	ECM
Il1b	Mmp2	Timp1	Fga
Il10	Mmp3	Timp2	Fgb
Il6	Mmp7	Timp3	Fgg
Tnf	Mmp8	Timp4	Fn1
Tgfb1	Mmp9	**Other proteases**	Vtn
Tgfb2	Mmp10	Tmprss6	Col11a1
Tgfb3	Mmp11	Tmprss7	Col1a1
**Reference genes**	Mmp12	Mcpt4	Col2a1
Ywhaz	Mmp13	Ctsg	Col3a1
Tbp	Mmp14	Elane	Col4a1
Gapdh	Mmp15	Hgfac	Col5a1
Rn18s	Mmp19	Prss8	Lama1
**PA-system**	Mmp21	Tpsb2	Lama2
Plat	Mmp23	St14	Lama4
Plau	Mmp24	**Protease inhibitors**	Lama5
Plaur	Mmp25	Spint1	Lamb1-1
Plg	Mmp27	Spint2	Lamb2
Serpinb2	Mmp28		Lamb3
Serpine1			Lamc1
Serpinf2			Lamc2
A2m			Lamc3

Table S1. qPCR array setup
Primer pairs specific for the listed genes were included on the StellARray^{TM}.
