# Supplementary material for: LPS Counter Regulates RNA Expression of Extracellular Proteases and Their Inhibitors in Murine Macrophages
Source: Mediators Inflamm. 2012 Mar 14;2012:157894. doi: 10.1155/2012/157894 (PMC3317238; doi:10.1155/2012/157894)
Supplement: Supplementary file 3 [file 157894.f3.pdf]

Expression normalized to DNA X 1000

MMPs	Ctrl	Vehicle			LPS		
Gene name	0 hours	2 hours	6 hours	18 hours	2 hours	6 hours	18 hours
Mmp2	32	27	29	36	47	47	62
Mmp3	ND	ND	ND	ND	3.2	4.1	6.7
Mmp7	ND	ND	ND	ND	2.6	2.4	2.7
Mmp8	31	31	22	23	35	27	44
Mmp9	889	822	601	371	929	904	1770
Mmp10	90	100	95	68	194	232	70
Mmp12	21094	25928	33442	38757	37947	56157	61261
Mmp13	562	610	546	272	1313	2264	1029
Mmp14	ND	ND	ND	ND	10.3	12.8	5.9
Mmp15	5.2	3.9	2.8	2.7	4.7	7.5	6.0
Mmp19	27	33	36	49	27	22	48
Mmp21	2.6	2.7	2.7	3.5	5.1	5.4	5.1
Mmp23	7.9	10.0	8.0	8.8	13.1	6.3	7.7
Mmp24	7.1	4.3	7.2	4.1	7.1	6.6	7.5
Mmp25	4.3	4.6	3.9	5.5	10.5	30.9	14.6
Mmp27	20	17	15	8	21	17	18
Mmp28	2.2	0.9	2.8	1.7	4.6	4.8	4.9

Cytokines

Il10	11.5	9.5	7.2	7.9	16.6	13.2	12.1
Il1b	13.1	4.5	3.3	6.0	1329	2871	1028
Il6	4.3	6.1	2.1	5.3	20	895	1787
Tnf	3367	2772	2936	3469	66111	45559	16787
Tgfb1	5889	5554	5423	4175	6238	5647	4300
Tgfb2	5.2	3.8	3.2	3.7	7.5	8.8	8.5
Tgfb3	67	57	60	57	66	42	28

PA-system

Plat	7.8	7.9	14.6	13.8	13.6	4.3	7.2
Plau	3607	3834	2861	2913	4598	847	941
Plaur	3961	3474	3181	4703	5937	7046	8504
Serpinb2	ND	ND	ND	ND	8.9	11.4	31.6
Serpine1	10	13	21	33	239	272	75
Serpinf2	ND	2.7	ND	3.3	ND	5.2	5.7
A2m	1.6	0.7	1.9	0.6	4.5	3.0	5.1

MMP inhibitors

Timp1	27	25	35	39	26	27	27
Timp2	3452	3502	3466	2776	3592	2147	1230
Timp4	3.4	2.6	ND	4.7	7.2	4.6	3.4

ECM

Fn1	18.0	18.0	11.6	3.5	18.9	11.8	ND
Vtn	3.1	2.4	ND	2.1	9.0	5.5	5.6
Col4a1	ND	2.9	ND	12.8	5.4	ND	16.1
Lama2	1.2	ND	0.9	ND	2.5	1.4	3.4
Lama5	13.2	12.2	17.5	14.1	19.8	18.8	26.3
Lamb2	5.2	4.7	3.5	4.6	5.1	6.9	8.2
Lamb3	ND	ND	ND	ND	5.2	14.3	30.4
Lamc1	1207	1105	1226	1128	1113	1108	1016

Other proteases

Tmprss6	5.2	4.2	6.0	3.1	9.6	6.2	6.8
Mcpt4	ND	ND	ND	ND	1.8	ND	2.1
Prss8	13.5	14.6	14.6	17.7	15.0	9.9	18.2
St14	1258	1375	1370	1405	1197	800	628

Table S2. Relative expression levels of analyzed genes
Gene expression levels were normalized to DNA as described thus allowing intergenic comparison.
