# Supplementary material for: LPS Counter Regulates RNA Expression of Extracellular Proteases and Their Inhibitors in Murine Macrophages
Source: Mediators Inflamm. 2012 Mar 14;2012:157894. doi: 10.1155/2012/157894 (PMC3317238; doi:10.1155/2012/157894)
Supplement: Supplementary file 4 [file 157894.f4.pdf]

Name	2 hours		6 hours		18 hours	
	Fold	p-value	Fold	p-value	Fold	p-value
**Cytokines**						
Il10	1.6	0.1036	1.4	0.0219	0.9	0.0471
Il1b	270.2	0.0000	643.5	0.0000	94.0	0.0004
Il6	2.2	0.1195	306.2	0.0000	198.9	0.0003
Tnf	21.7	0.0000	11.5	0.0000	2.7	0.0009
Tgfb1	1.0	0.5208	0.8	0.0323	0.6	0.0240
Tgfb2	1.7	0.0216	2.2	0.0221	1.3	0.1709
Tgfb3	0.8	0.2853	0.5	0.0092	0.3	0.0032
**Reference genes**						
Ywhaz	0.9	0.2593	0.8	0.0134	0.5	0.0093
Tbp	0.9	0.3524	0.8	0.0128	0.5	0.0125
Gapdh	0.9	0.4441	0.7	0.0077	0.7	0.0183
Rn18s	0.8	0.3396	0.6	0.0330	0.7	0.0325
**PA-system**						
Plat	1.1	0.2822	0.2	0.0018	0.3	0.0145
Plau	1.1	0.5212	0.2	0.0001	0.2	0.0003
Plaur	1.5	0.0470	1.7	0.0016	1.0	0.0122
Serpinb2	ND	0.0115	ND	0.0042	ND	0.0001
Serpine1	16.9	0.0003	9.5	0.0003	1.3	0.0279
Serpinf2	ND	0.3741	ND	0.0064	1.0	0.1200
A2m	5.3	0.0142	1.2	ND	5.0	0.0057
**Extracellular matrix**						
Fn1	0.7	0.3193	0.8	0.0729	ND	0.1305
Vtn	3.3	0.0283	ND	0.0334	1.4	0.1807
Col4a1	1.2	0.1932	ND	ND	0.7	0.0309
Lama2	ND	0.0565	1.1	ND	ND	0.0302
Lama5	1.1	0.2423	0.8	0.0615	1.1	0.0554
Lamb2	0.7	0.3647	1.5	0.0224	1.0	0.0880
Lamb3	ND	0.0072	ND	0.0023	ND	0.0001
Lamc1	0.7	0.2474	0.7	0.0159	0.5	0.0172

Name	2 hours		6 hours		18 hours	
	Fold	p-value	Fold	p-value	Fold	p-value
**MMPs**						
Mmp2	1.6	0.0638	1.2	0.0054	5.0	0.0320
Mmp3	ND	0.0168	ND	0.0077	ND	0.0243
Mmp7	ND	0.1103	ND	ND	ND	ND
Mmp8	1.0	0.4618	0.9	0.0075	1.0	0.0165
Mmp9	1.0	0.5390	1.1	0.0098	2.6	0.0007
Mmp10	1.8	0.0262	1.8	0.0024	0.6	0.0267
Mmp12	1.3	0.1119	1.2	0.0099	-1.1	0.0419
Mmp13	1.9	0.0081	3.1	0.0004	2.1	0.0024
Mmp14	ND	0.0003	ND	0.0022	ND	ND
Mmp15	0.7	0.5086	2.0	0.0038	1.2	0.1824
Mmp19	0.7	0.0884	0.4	0.0038	0.5	0.0342
Mmp21	1.3	0.1705	1.6	0.0240	0.9	0.2316
Mmp23	0.9	0.2835	0.6	0.0328	0.5	0.0691
Mmp24	1.1	0.3804	0.7	0.1875	1.0	0.1279
Mmp25	2.0	0.0162	5.8	0.0023	1.4	0.0067
Mmp27	0.9	0.3145	0.8	0.0136	1.2	0.0312
Mmp28	4.6	0.0009	1.3	0.0999	1.6	0.0291
**Inhibitors of MMPs**						
Timp1	0.7	0.2266	0.6	0.0090	0.4	0.0275
Timp2	0.7	0.2299	0.5	0.0028	0.2	0.0013
Timp4	1.9	0.1254	ND	0.1181	0.4	0.0867
**Other proteases**						
Tmprss6	1.5	0.1704	0.8	0.0509	1.2	0.1996
Mcpt4	ND	0.0010	ND	0.0049	ND	0.0344
Prss8	0.9	0.4646	0.5	0.0226	0.6	0.0294
St14	0.8	0.1008	0.4	0.0034	0.2	0.0011

Table S3. GPR fold change values and GPR p-values of array data

Gene expression in LPS and vehicle stimulated RAW 264.7 cultures was analyzed after 2, 6, and 18 hours. The GPR fold change (Fold) is presented and GPR p-values (p-value) are highlighted in red when below 0.05. The GPR fold change values are provided in this table to indicate how the gene is regulated, but it should be stressed that the GPR p-values are not directly linked to the fold change values.
